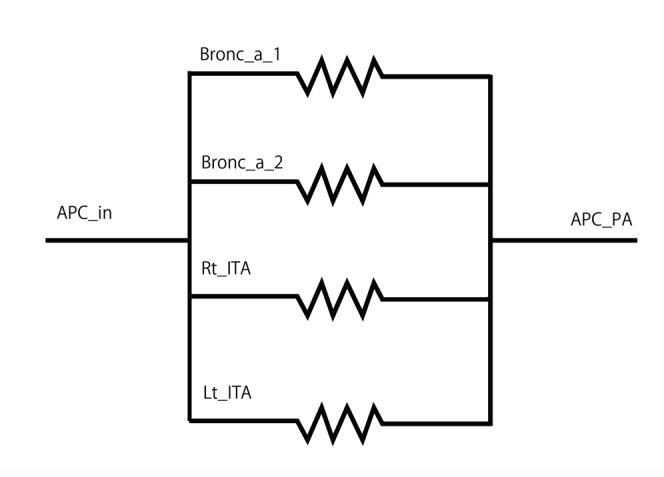# Impact of Collateral Vessels on Fontan Circulation: 0- to 1-Dimensional Fontan Circulation Model and Concept of Oxygen Supply and Consumption

**DOI:** 10.1016/j.atssr.2025.01.003

**Published:** 2025-01-31

**Authors:** Koichi Sughimoto, Toru Miki, Ruichen Li, Kenshu Maeda, Daiki Koda, Takashi Fujiwara, Hao Liu

**Affiliations:** 1 Graduate School of Engineering, Chiba University, Japan; 2 Section of Pediatric Radiology, Department of Radiology, Children's Hospital Colorado, University of Colorado Anschutz Medical Campus, Aurora, Colorado

## Abstract

**Background:**

Patients with single ventricle often undergo the Fontan operation to alleviate cyanosis and reduce ventricle volume load. However, long-term outcomes are limited by complications and hemodynamic issues, including collateral vessels, leading to cyanosis and heart failure. Studies have demonstrated considerable blood flow through these collateral vessels; however, their hemodynamic impact on Fontan circulation remains inadequately explained from a theoretical perspective. This study aims to clarify the effects of dobutamine and vasodilators on Fontan circulation in the presence of aortopulmonary collaterals (APCs) and venovenous collaterals (VVCs).

**Methods:**

A Fontan hemodynamic model incorporating VVC and APC was created. Scenarios were simulated by adjusting dobutamine dosage and oxygen delivery/consumption, predicting arterial and central venous oxygen saturations. Cardiac function was evaluated based on cardiac output, arterial elastance, ejection fraction, and stroke work to pressure-volume ratio.

**Results:**

Fontan circulations with VVC and APC had lower arterial and venous oxygen saturations (92% and 54%, respectively) compared with those without collaterals (96% and 62%, respectively). Decreased arterial elastance and increased stroke work to pressure-volume ratio indicated poor tissue perfusion. High pulmonary resistance decreased oxygen saturations and systemic blood flow, regardless of collaterals. Dobutamine (10 μg/kg/min) raised venous oxygen from 53% to 58%, respectively, in the presence of VVC and APC, but decreased arterial oxygen from 92% to 88%, respectively.

**Conclusions:**

The results align with clinical findings, suggesting pulmonary vasodilators may improve oxygenation and perfusion in Fontan patients with collaterals. However, dobutamine effects are limited. Validation with actual patient data is needed to enhance model accuracy.

## Visual Abstract

**Figure:**
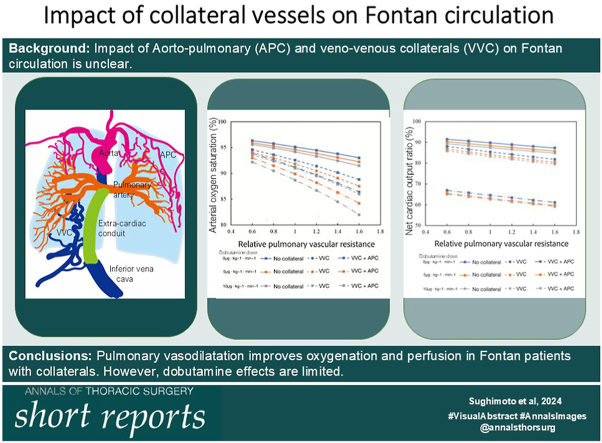

**In Short**

▪ Collateral vessels, such as aortopulmonary and venovenous collaterals, have a particularly detrimental impact on failing Fontan circulations.
▪ Dobutamine shows limited efficacy in improving hemodynamics when collateral vessels coexist in Fontan circulation.
▪ There are potential benefits of pulmonary vasodilators in improving Fontan circulation hemodynamics, even in the presence of collateral vessels.

Since Francis Fontan's initial report in the 1970s,^1^ the Fontan operation has significantly contributed to saving the lives of children with a single ventricle. Advances in surgical strategies, methodologies, and the introduction of progressive medical treatments have enhanced the long-term survival rates post-Fontan operation, which now range between 70% and 80%.^2^

Despite these notable improvements, individuals with the Fontan circulation often encounter various complications including arrhythmias, thrombus formation along the pathway, desaturation, and protein-losing enteropathy, ultimately leading to heart failure. Collateral vessels may contribute to and exacerbate these clinical conditions. Nevertheless, there remains a paucity of information regarding the impact of collateral vessel development on the hemodynamics of Fontan circulation.

This study aims to elucidate the influence of dobutamine and pulmonary vasodilators on Fontan circulation in the presence of 2 types of collateral vessels: aortopulmonary collaterals (APCs) and venovenous collaterals (VVCs). Additionally, the study seeks to predict arterial and venous oxygen saturation levels by introducing the concept of oxygen delivery and consumption. Furthermore, various parameters of cardiac function will be predicted using a 0- to 1-dimensional Fontan circulation hemodynamic computational fluid dynamic model.

## Material and Methods

### Lumped Parameter Model of Fontan Circulation

**Supplementary Figure 1:**
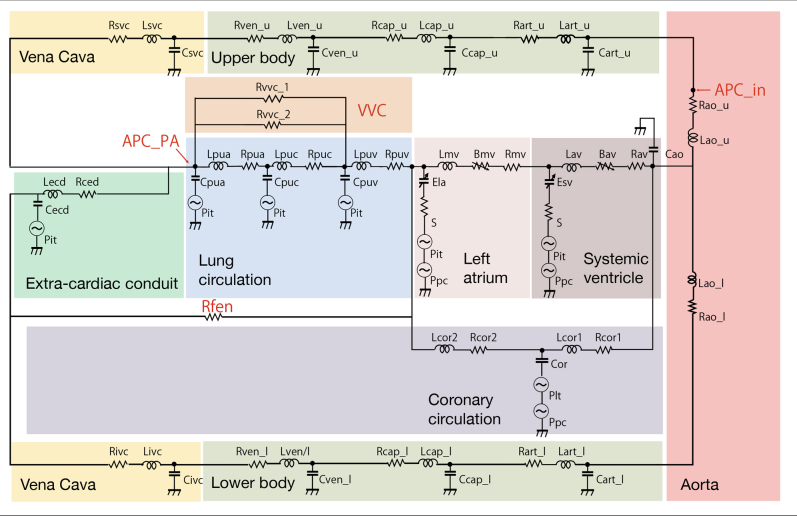


This study followed our previous method, utilizing a lumped parameter model of the Fontan cardiovascular system comprising a time-varying elastance chamber component and a Windkessel vascular component to simulate heart dynamics and hemodynamics in the arterial and venous trees.^3^^,^^4^ This model encompasses the heart, pulmonary, and systemic circulations; systemic organs; upper and lower extremities; and coronary circulation. Specifically designed for patients with a single systemic left ventricle, the model integrates parameter optimization, clinical data-matched model development, and sensitivity analysis–based parameter selection. The model redirects venous flow from the lower body to the right pulmonary artery, as described in detail in a separate article.^4^, ^5^, ^6^ Each compartment was modeled using three elements: viscous resistance, vessel compliance, and inertial resistance. The heart chambers were characterized by viscoelasticity coefficients and time-varying elastances to represent their contractility. In the total cavopulmonary connection Fontan model, both the superior and inferior vena cavae were connected to the pulmonary artery, with an artificial graft inserted between the inferior vena cava and pulmonary artery. Additionally, fenestration was established between the conduit and atrium in the total cavopulmonary connection model, as described previously.^4^ We developed idealized computational models based on our previous study, where the patient’s body surface area was given as 1.63 m^2^. A 0- to 1-dimensional Fontan circulation hemodynamic model was developed to simulate blood flow changes throughout the body. This model incorporates two types of collateral vessels: VVCs and APCs (Supplemental Figures 1, 2), considering clinical conditions. VVCs connect systemic veins with desaturated blood to pulmonary veins, reducing pulmonary vein saturation. APCs, such as mammary arteries, link systemic arteries to pulmonary arteries, resulting in increased saturation in the pulmonary artery due to the pressure gradient. The model investigates different conditions and makes predictions by altering dobutamine dosage and integrating concepts of oxygen delivery and consumption through changes in workload. The effects of dobutamine were simulated using the approach outlined in our recent method,^3^ which was based on previous studies.^5^ In these studies, parameters such as end-systolic ventricular elastance and systemic vascular resistance were modified according to the dobutamine infusion rate. For this report, we applied the same methodology. The standard values for systemic and pulmonary vascular resistance were set at 0.9 and 0.05 mm Hg.s/mL, respectively, as reported in our previous publication.^6^ The phenomena were simulated by adjusting the systemic and pulmonary resistance, scaling them with magnification ratios ranging from 0.6 to 1.6.

Predictions include arterial and central venous oxygen saturations (SaO_2_ and SvO_2_) in the Fontan circulation models.

The equations defining oxygen consumption index (VO₂I), cardiac output index (COI), and arterial and venous oxygen content (CaO₂, CvO₂) are based on the Fick principle:

$$VO_{2}I=COI\;\times \;\left(CaO_{2}-CvO_{2}\right)\times 10$$

$$CaO_{2}=Hb\times 1.36\;\times \;\frac{SaO_{2}}{100}\;+\;PaO_{2\;}\times 0.0031$$

$$CvO_{2}=Hb\times 1.36\;\times \;\frac{SvO_{2}}{100}\;+\;PvO_{2\;}\times 0.0031$$

In these equations, the hemoglobin level (Hb) is assumed to be 15 g/dL. The initial arterial and venous oxygen saturations are given as 97% and 60%, respectively.

To evaluate cardiac function, considering the impact of collateral vessels, 4 representative values were assessed: (1) cardiac output per minute; (2) end-systolic elastance (Ees) relative to arterial elastance (Ea) (Ees/Ea); (3) ejection fraction (EF); and (4) the stroke work (SW) to pressure-volume area (PVA) ratio (SW/PVA).

We affirm that all procedures involved in this study adhere to the ethical standards outlined in the relevant national guidelines for human experimentation (Ethical Guidelines for Medical and Health Research Involving Human Subjects, Ministry of Health, Labor and Welfare, 2003) and comply with the Helsinki Declaration of 1975, as revised in 2008.

## Results

### Systemic Arterial and Venous Oxygen Saturation

**Figure 1:**
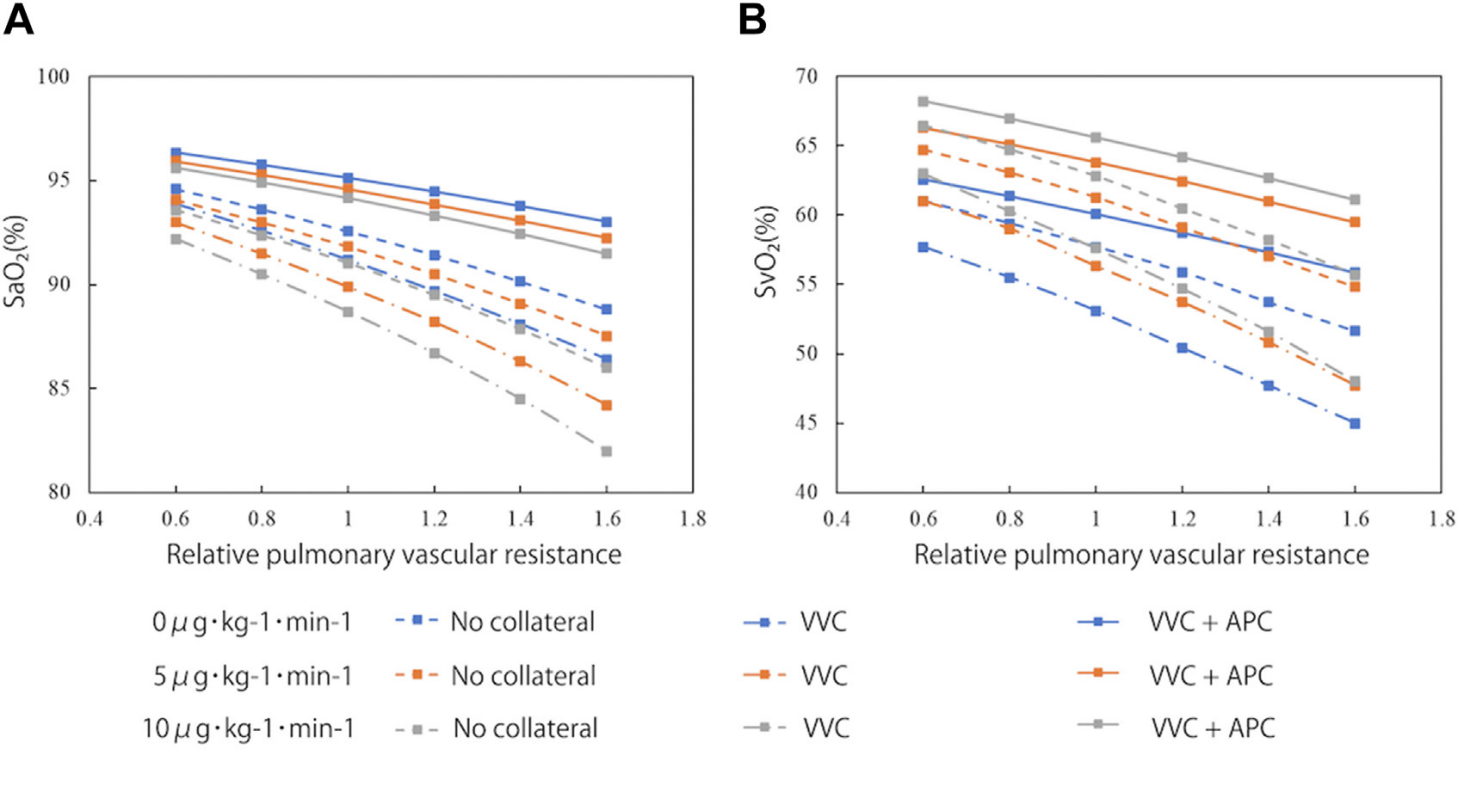
(A) Systemic arterial oxygen saturation in various pulmonary vascular conditions and different dose of dobutamine. (B) Systemic venous oxygen saturation in various pulmonary vascular conditions and different dose of dobutamine. (APC, aortopulmonary collateral; SaO_2_, arterial oxygen saturation; SvO_2_, venous oxygen saturation; VVC, venovenous collateral.)

Systemic arterial and venous oxygen saturation were calculated under various conditions by incorporating the concept of oxygen delivery and consumption (Figure 1). Elevated pulmonary vascular resistance led to a decrease in systemic arterial oxygen saturation. The presence of VVCs resulted in reduced systemic arterial oxygen saturation, while coexistence of VVCs and APCs lowered systemic arterial oxygen saturation (96% vs 92%; without vs with collaterals at the baseline pulmonary vascular resistance). Administration of dobutamine did not improve systemic arterial oxygen saturation and actually decreased it (eg, from 92% to 88% at the baseline pulmonary vascular resistance). For systemic venous oxygen saturation, the presence of VVCs decreased systemic venous oxygen saturation, and when VVCs and APCs coexisted, systemic venous oxygen saturation decreased more significantly (62% vs 54%; with vs without collaterals at the baseline pulmonary vascular resistance). Administration of a higher dose of dobutamine increased systemic venous oxygen saturation (eg, from 53% to 58% at the baseline pulmonary vascular resistance).

### Cardiac Output and Net Cardiac Output

**Figure 2:**
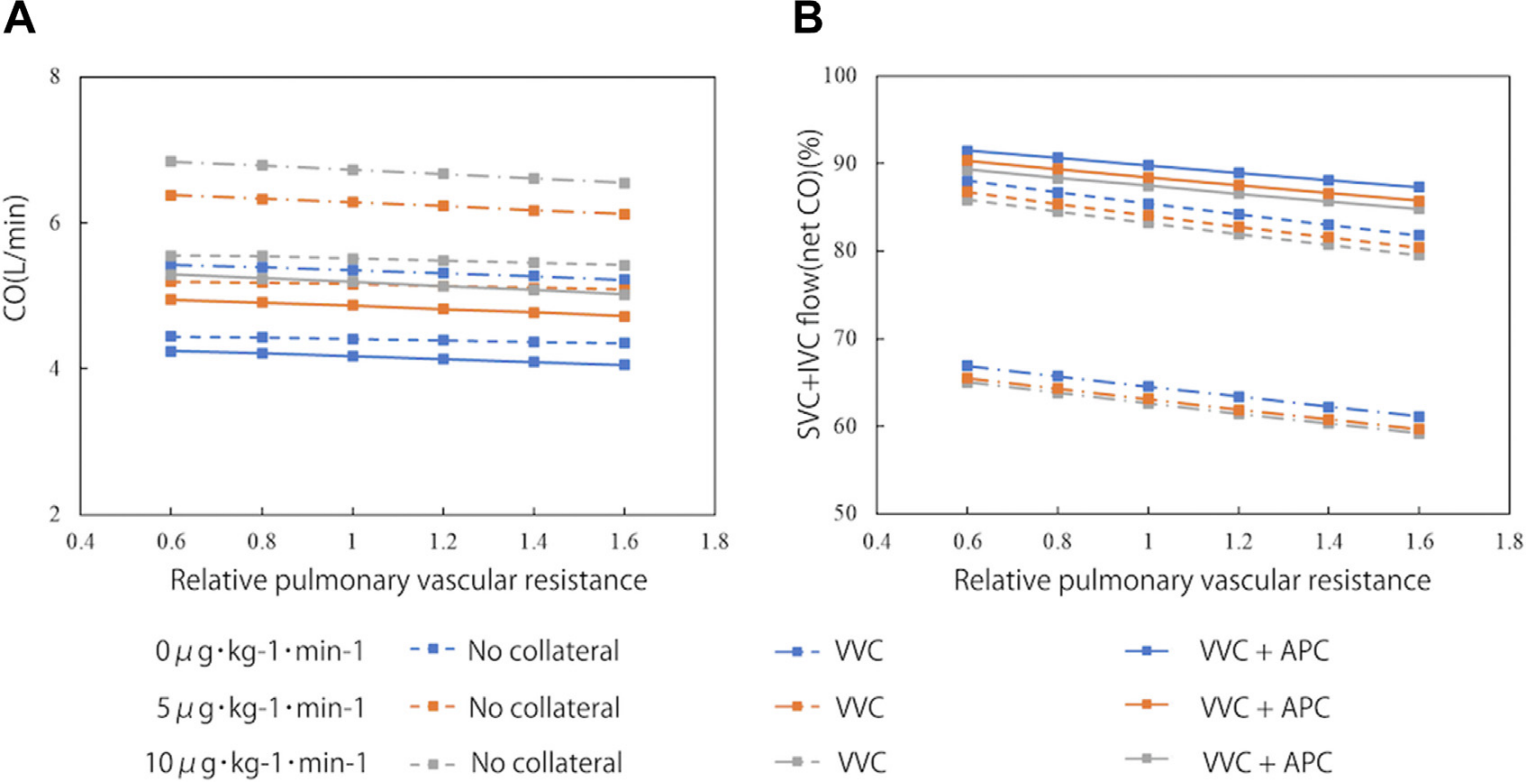
(A) Cardiac output in various pulmonary vascular conditions and different dose of dobutamine. (B) Net cardiac output in various pulmonary vascular conditions and different dose of dobutamine. (APC, aortopulmonary collateral; CO, cardiac output; IVC, inferior vena cava; SVC, superior vena cava; VVC, venovenous collateral.)

Elevated pulmonary vascular resistance decreased cardiac output, with VVCs augmenting it (Figure 2). When VVC and APC coexisted, cardiac output further increased. Higher doses of dobutamine also increased cardiac output. Net cardiac output, defined as blood returning from both superior and inferior vena cavae to supply organs and tissues throughout the body, decreased with higher pulmonary vascular resistance. Administration of dobutamine did not improve but reduced net cardiac output, which was more remarkable under coexistence of VVCs and APCs.

### Pressure-Volume Curves

**Figure 3:**
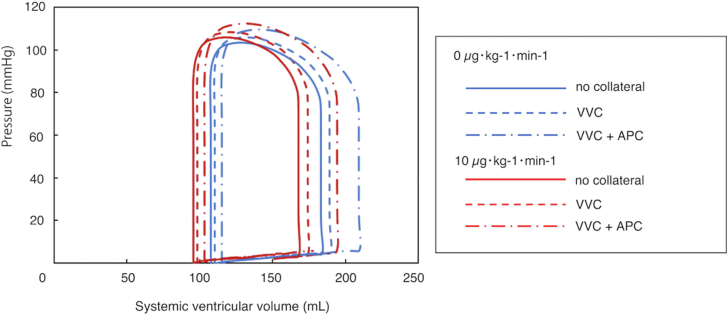
Pressure volume curve of the systemic ventricle of Fontan circulation. (APC, aortopulmonary collateral; VVC, venovenous collateral.)

Pressure-volume curves of the systemic ventricle were plotted under various conditions. Administration of dobutamine shifted the curve to the left, indicating increased contractility. Conversely, VVCs expanded the curve to the right, while the coexistence of VVCs and APCs further increased the curve area and shifted it more rightward, suggesting increased preload and decreased contractility (Figure 3).

### Cardiac Function

**Figure 4:**
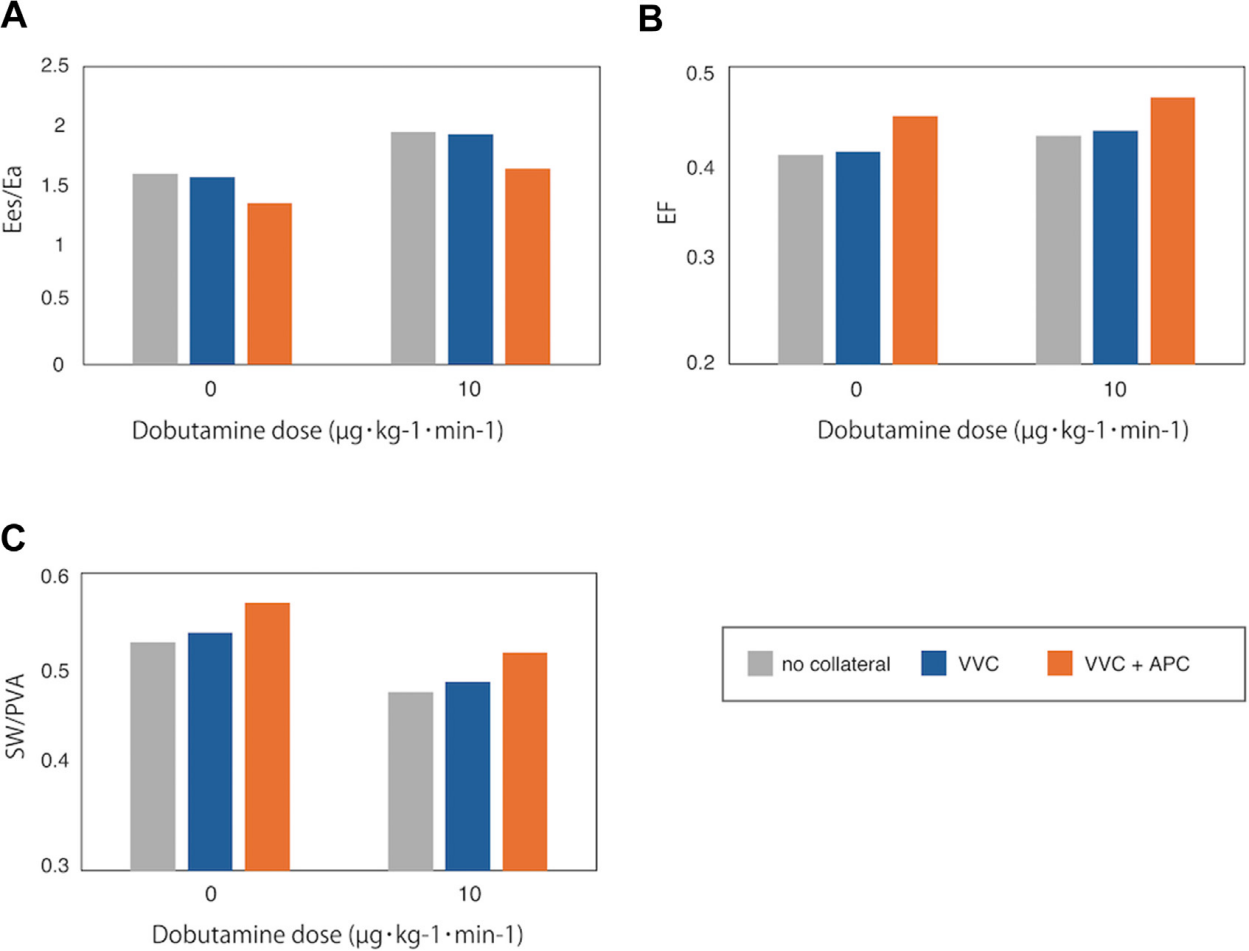
(A-C) Dobutamine administration trends. (APC, aortopulmonary collateral; EF, ejection fraction; Ees/Ea, relative end-systolic ventricular elastance to arterial elastance; SW/PVA, stroke work per pressure volume area; VVC, venovenous collateral.)

Three indices, EF, Ees/Ea, and SW/PVA, were computed under different conditions. EF was enhanced by VVCs and the coexistence of VVCs and APCs, with slight elevation upon dobutamine administration. In terms of Ees/Ea, dobutamine administration increased Ees/Ea, but VVCs decreased Ees/Ea, further decreasing when VVCs and APCs coexisted. Regarding SW/PVA, dobutamine administration reduced SW/PVA, while VVCs and the coexistence of VVCs and APCs increased SW/PVA. These trends are illustrated in Figure 4.

## Comment

In Fontan circulation, 2 types of collateral vessels typically form between the systemic and pulmonary circulations, namely APCs and VVCs. APCs, originally recognized as mammary arteries, bronchial arteries, and intercostal arteries, are vessels connecting the aorta with major arteries and the pulmonary artery.^7^ These vessels develop in size, particularly in cases of cyanosis, forming abnormal connections with the pulmonary arteries. These APCs naturally augment blood flow to the pulmonary artery, thereby improving cyanosis. Cardiac magnetic resonance imaging studies have shown that, in single ventricle patients prior to Fontan operation, these collaterals contribute to 40% to 60% of pulmonary blood flow.^8^ APCs increase cardiac output by increasing blood returning to the systemic ventricle, consequently elevating preload on the systemic ventricle. However, especially in cases involving a naive single ventricle, such as the anatomical right ventricle or a single ventricle with a common atrioventricular valve, these ventricles may not be able to cope with the increased preload, potentially leading to Fontan heart failure. On the contrary, VVCs typically originate from the lower body veins with increased venous pressure, connecting either to branches of the pulmonary veins or directly to the left atrium, a lower-pressure chamber. These collaterals, containing desaturated blood, exacerbate cyanosis in the systemic artery. Nevertheless, they serve a role akin to fenestration in Fontan circulation, facilitating venous flow return to the left chambers without traversing the pulmonary capillary arteries' high resistance, thus enhancing cardiac output while sacrificing desaturation. Despite their influence on Fontan circulation, there has been a lack of theoretical understanding. This study aimed to provide significant insights into Fontan circulation involving these collaterals. The present study found that when both APCs and VVCs coexist—which is common among older patients with Fontan circulation—systemic arterial and venous oxygen saturation significantly decrease. Surgical elimination of APCs, such as by clipping mammary arteries or ligating collateral vessels, is meaningful for improving the hemodynamics of Fontan circulation. Preoperative catheter interventions, including coiling in mammary arteries^9^ and VVCs,^10^ also yield positive effects; however, they may have limited efficacy due to the development of other collaterals in the early and late phases postintervention.

In the postoperative period, dobutamine's impact on Fontan circulation is limited, with dobutamine showing a positive effect on Eea/Ea, while SW/PVA decreases with increased dobutamine dosage. Despite traditional intensive care unit strategies to enhance hemodynamics, higher dobutamine doses may not necessarily increase cardiac output, and, from an energy efficiency perspective, SW/PVA worsens with increased dobutamine dosage, particularly evident when collateral vessels are present in Fontan circulation. Although the effect of pulmonary vasodilators on Fontan circulation was not directly tested in this study, the use of drugs such as nitric oxide, sildenafil, and oxygen, which reduce pulmonary vascular resistance, may positively influence cardiac output as well as arterial and venous oxygen saturation in Fontan circulation. Lowering pulmonary vascular resistance is crucial for improving Fontan circulation, especially when collateral vessels are present, making these pulmonary vasodilators important.

In conclusion, simulated results indicate that pulmonary vasodilators could potentially enhance blood oxygen saturation and tissue perfusion, even in Fontan patients with collateral vessels. This beneficial effect persists despite the limited influence of dobutamines on cardiac function. Validation of predicted values through actual patient data collection is important to enhance model credibility for reproducing various Fontan circulation conditions.

## Figures and Tables

**Supplementary Figure 2:**