# Supplementary material for: Impact of Collateral Vessels on Fontan Circulation: 0- to 1-Dimensional Fontan Circulation Model and Concept of Oxygen Supply and Consumption
Source: Ann Thorac Surg Short Rep. 2025 Jan 31;3(3):657–62. doi: 10.1016/j.atssr.2025.01.003 (PMC12559264; doi:10.1016/j.atssr.2025.01.003)
Supplement: Supplementary Figures legends [file mmc1.docx]

**Supplemental Figure 1**

Schematic diagram of lumped parameter model of Fontan circulation with collateral vessels.

APC_in: aorto-pulmonary collateral inlet; APC_PA: aorto-pulmonary collateral’s pulmonary artery side; Rfen: fenestration resistance; VVC: veno-venous collaterals

B: Bernoulli resistance; C: compliance; E: elastance; L:inertance; Pit: intrathoracic pressure; Ppc: pericardial pressure; R: viscous resistance; S: viscoelasticity coefficient.

Subscripts

ao: aorta; apc: art: arteries; av: aortic valve; cap: capillaryes; cor: coronary circulation; ivc: inferior vena cava; l: lower body; mv: mitral valve; pua: pulmonary arteries; puc: pulmonary capillaries; puv: pulmonary veins; shu: shunt of pulmonary veno-venous fistula; svc: superior vena cava; u: upper body; ven: vein

**Supplemental Figure 2**

Schematic diagram of aorto-pulmonary collaterals.

APC in: aortopulmonary collateral inlet; APC_PA: aorto-pulmonary collateral’s pulmonary artery side; Bronc_a: bronchial artery; Lt_ITA: left internal thoracic artery; Rt_ITA: right internal thoracic artery
